# Sirolimus (Rapamycin) Induced Mucosal Healing in Anti-Tumor Necrosis Factor Refractory Pediatric Ulcerative Colitis

**DOI:** 10.1097/PG9.0000000000000183

**Published:** 2022-03-17

**Authors:** Richard Kellermayer, Andrew Chang, Kalyani Patel

**Affiliations:** * Section of Pediatric Gastroenterology, Texas Children’s Hospital Baylor College of Medicine; † USDA/ARS Children’s Nutrition Research Center; ‡ Baylor College of Medicine; § Department of Pathology, Baylor College of Medicine, Houston, TX.

## Abstract

Sirolimus (rapamycin) has been sparsely reported in the treatment of pediatric ulcerative colitis (PUC). Mucosal healing has not been examined in responders to the drug. We describe a case of infliximab refractory PUC where rapamycin induced sustained clinical remission along with mucosal healing. We conclude that rapamycin should be positioned into the expanding treatment repertoire of PUC.

## INTRODUCTION

About 6% of patients with pediatric ulcerative colitis (PUC) progress to colectomy within 1 year (1), and around 13% by 5 years (2) of diagnosis. Current treatment guidelines for steroid dependent or refractory, moderate-to-severe disease call for infliximab (IFX) as the primary anti-tumor necrosis factor (TNF) biologic therapy of choice (3). Consideration for colectomy is recommended after anti-TNF failure (3). Alternative rescue treatments include tacrolimus and cyclosporine (3), but sirolimus (rapamycin) has only been proposed as a promising option in a small case series (4). Endoscopic assessment of responders was not performed in that work. Here we report on an anti-TNF refractory PUC case, where sirolimus treatment brought mucosal healing with sustained clinical and biochemical remission for over 15 months.

## CASE REPORT

**FIGURE 1.**
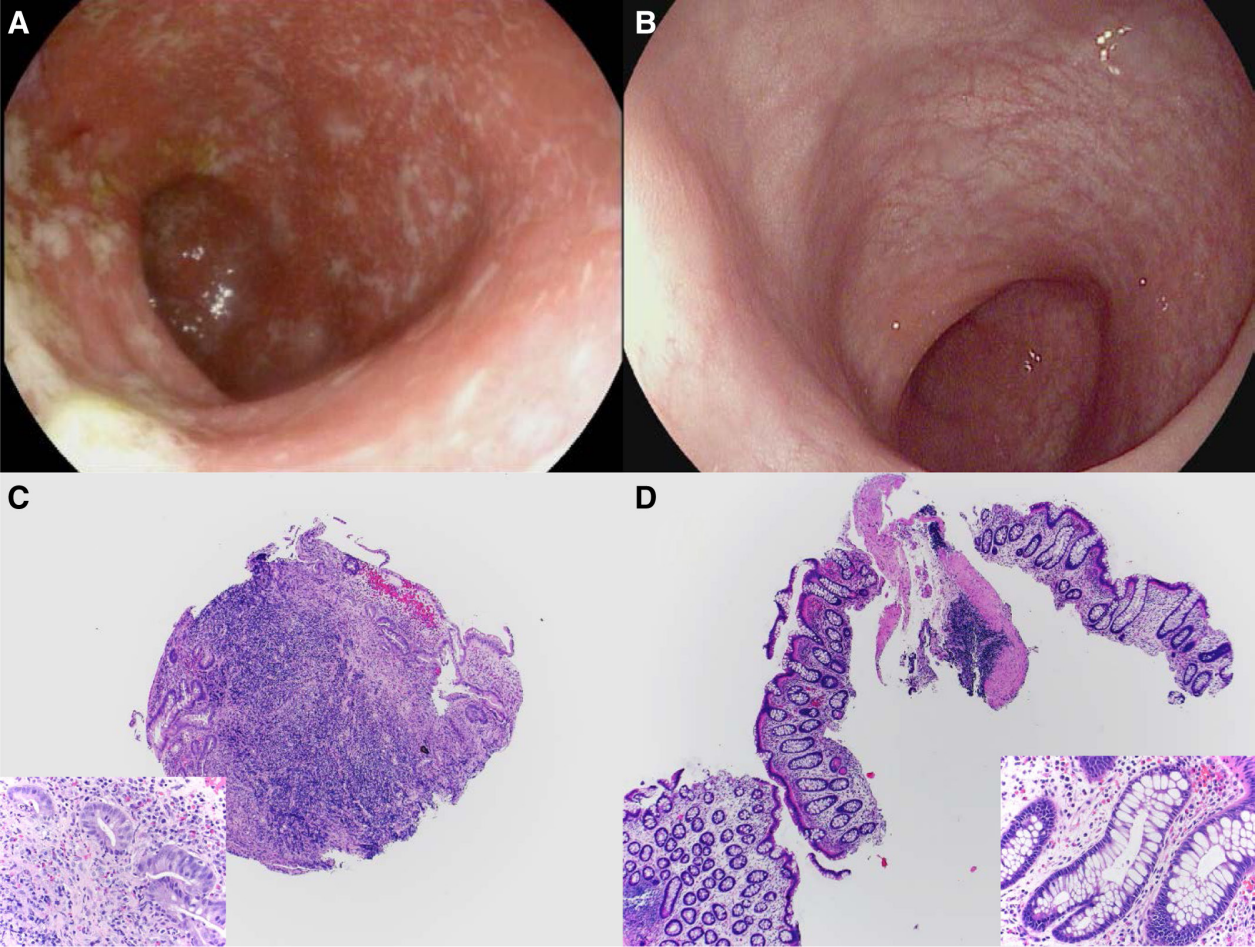
Endoscopic and histologic images from the descending colon. A) Moderate colitis on infliximab therapy. B) Endoscopic healing on sirolimus monotherapy. C) Descending colon biopsy on infliximab therapy: single piece of colonic mucosa with marked chronic active inflammation, crypt distortion, and crypt loss (H&E, ×40 magnification); insert: cellular lamina propria is seen with neutrophilic infiltration of the crypt epithelium along with prominent eosinophils (×400). D) Descending colon biopsy: multiple pieces of colonic mucosa show sparse crypt branching without other signs of inflammation (H&E, ×40 magnification); insert: lamina propria is paucicellular, no increased neutrophils or eosinophils are seen; there is no acute cryptitis (×400). H&E = hematoxylin and eosin.

A 12-year-old male with maternal history of ulcerative colitis and personal history of isolated left leg lymphangiectasia presented after 6 weeks of diarrhea and hematochezia. Endoscopy-based diagnosis of moderately active pancolitis (Paris E4) was made. He responded well to systemic steroids, but recurrence occurred during his wean, and IFX therapy was initiated 3 months after diagnosis. He could not achieve clinical remission on IFX, in spite of intensification to every 4-week 7.5 mg/kg dosing and reaching a maintenance level of 5.4 mcg/mL. Fecal calprotectin (FC) remained above 900 mcg/g (normal, <126 mcg/g). Intensified dosing of IFX was initiated at 10 mg/kg, 3 weeks after the infusion where the trough level was obtained. Three weeks after this intensified dose, however, a moderate clinical flare occurred when FC rose to 7990 mcg/g, and the patient and family requested other treatment options. Colonoscopy then 1 year into diagnosis showed moderate (Mayo 2) pancolitis (Fig. 1A, C). After reviewing alternative treatment options, sirolimus was chosen with hopes that it may have beneficial effects on the left leg lymphangiectasia (5) as well. He was started on 37.5 mcg/kg sirolimus with a goal level of 8 to 10 ng/mL (4). During dose adjustments, his sirolimus levels varied between 3.2 and 13.6 ng/mL. He achieved rapid clinical response within 5 to 6 days of initiation and clinical remission by 2 months with an FC of 205 mcg/kg. The patient ended up on 125 mcg/kg daily maintenance dosing with levels between 7.7 and 9.7 ng/mL and FC of 51 mcg/g by 3.5 months into treatment. He gained weight and height and remained in clinical remission for over 17 months to date, in spite of drug levels declining to 3 ng/mL at 100 mcg/kg dosing, which was deliberately allowed due to his good condition. A follow-up colonoscopy 8 months after starting sirolimus showed mucosal healing by endoscopy and histology (Fig. 1B, D). FC after 14 months of treatment was 71 mcg/g. Liver function and lipid panel tests remained within normal limits, and no adverse effects or events were noted. His leg lymphangiectasia was unaffected.

## DISCUSSION

PUC continues to be an aggressive disease with earlier progression to colectomy than in adult ulcerative colitis. Our case indicates that further clinical research could position sirolimus in the expanding biologic and small molecule treatment repertoire for PUC. As in prior reports (4), sirolimus was safe in our patient for over 17 months. In the meantime, long-term monitoring for side effects including hypertension, thromboembolism, headaches, hypertriglyceridemia, constipation, acne, bone marrow suppression, and lymphomas is important.

## ACKNOWLEDGMENTS

The authors would like to acknowledge the Gutsy Kids Fund, including philanthropic leadership by the Brock Wagner family and donations from the Frugoni and other generous families (to R.K.); the Klaasmeyer family funds for PSC research (to R.K.); and the Crohn’s and Colitis Foundation ProKiids Network (for grant/research time support to R.K.). R.K. contributed to conceptual design, data collection, and manuscript writing; A.C. to data collection and manuscript writing; and K.P. to data collection, images, and manuscript writing.
